# The Indigenous Australian Human Papillomavirus (HPV) Cohort Study 2, Continuation for 5 to 10 Years: Protocol for a Longitudinal Study

**DOI:** 10.2196/44593

**Published:** 2023-05-17

**Authors:** Joanne Hedges, Sneha Sethi, Gail Garvey, Lisa J Whop, Karen Canfell, Zell Dodd, Priscilla Larkins, Annika Antonsson, Megan A Smith, Murthy Mittinty, Catherine Leane, Nicolas Reid, Eng H Ooi, Xiangqun Ju, Richard Logan, Lisa Jamieson

**Affiliations:** 1 Australian Research Centre for Population Oral Health Adelaide Dental School The University of Adelaide Adelaide Australia; 2 Epidemiology and Health Systems Menzies School of Health Research University of Queensland Brisbane Australia; 3 College of Health and Medicine Australian National University Canberra Australia; 4 The Daffodil Centre The University of Sydney, a joint venture with Cancer Council NSW Sydney Australia; 5 Yadu Health Aboriginal Corporation Ceduna Australia; 6 Umoona Tjutagku Health Service Aboriginal Corporation Coober Pedy Australia; 7 QIMR Berghofer Medical Research Institute Brisbane Australia; 8 Faculty of Medicine University of Queensland Brisbane Australia; 9 School of Public Health The University of Adelaide Adelaide Australia; 10 Strategic Partnerships Aboriginal Health Division Women's and Children's Health Network Adelaide Australia; 11 Nunkuwarrin Yunti of South Australia Adelaide Australia; 12 Flinders Medical Centre College of Medicine and Public Health Flinders University Adelaide Australia; 13 Adelaide Dental School The University of Adelaide Adelaide Australia

**Keywords:** Aboriginal South Australian, human papillomavirus, oral HPV infection, oral pharyngeal squamous cell carcinoma, OPSCC

## Abstract

**Background:**

Human papillomavirus (HPV) infection, a common sexually transmitted disease, is associated with cancers of the cervix, vulva, vagina, penis, anus, and head and neck. Oropharyngeal squamous cell carcinoma (OPSCC; throat cancer) is a type of cancer involving the head and neck area that is rapidly increasing across the globe. There are higher rates of OPSCC among Indigenous populations relative to non–Indigenous Australian populations, although the HPV-attributable fraction remains unknown. For the first time at a global level, we plan to extend an Indigenous Australian adult cohort to monitor, screen, and ultimately prevent HPV-associated OPSCC and to undertake extensive cost-effectiveness modelling around HPV vaccination.

**Objective:**

This study aims to (1) extend follow-up to a minimum of 7 years post recruitment to describe the prevalence, incidence, clearance, and persistence of oral HPV infection; and (2) conduct clinical examinations of the head and neck, oral cavity, and oropharynx and collect saliva samples for early-stage OPSCC testing.

**Methods:**

We will continue to implement a longitudinal design for the next study phase, where we will ascertain the prevalence, incidence, clearance, and persistence of oral HPV infection at 48, 60, and 72 months; undertake clinical examinations/saliva assessments to detect early-stage OPSCC; and refer for treatment. The primary outcome measures are changes in oral HPV infection status, biomarker measures of early HPV-related cancer, and clinical evidence of early-stage OPSCC.

**Results:**

Participant 48-month follow-up will commence in January 2023. The first results are expected to be submitted for publication 1 year after 48-month follow-up begins.

**Conclusions:**

Our findings have potential to change the way in which OPSCC among Australian Indigenous adults is managed, with desired impacts including cost-savings on expensive cancer treatments; improved nutritional, social, and emotional outcomes; and improved quality of life for both Indigenous adults and the Indigenous community more broadly. Continuing a large, representative Indigenous adult cohort to track oral HPV infection and monitor early OPSCC is essential to yield critical information to include in the management armamentarium of health and well-being recommendations for Australia’s First Nations.

**International Registered Report Identifier (IRRID):**

PRR1-10.2196/44593

## Introduction

### Human Papillomaviruses

Human papillomaviruses (HPVs) are a heterogeneous group of circular, double-stranded DNA viruses that grow in stratified epithelium (skin and mucosa). Prior to the implementation of vaccination, HPV was the most common sexually transmitted infection in Australia, with an estimated 4 out of 5 Australians having an HPV infection at some point in their lives [1]. HPV is a precursor to a range of cancers, including cervical cancer, other anogenital cancers, and oropharyngeal cancer. It is usually transmitted within 2-5 years of sexual activity debut [2]. The prevalence of HPV infection at a population level in Australia is unknown as there is no national level or representative survey data, and almost all surveys have focused on females only. There is reasonable HPV DNA data on prevaccination cervical HPV infection in females in both general and Indigenous populations. For example, prior to HPV vaccination, a survey of females aged 15-40 years attending routine Papanicolaou screening at 16 Indigenous health services, 8 family-planning services, and 10 community clinics sites across Australia estimated the age-standardized prevalence of any high-risk (oncogenic) HPV type as 31.3% and 30% among 655 Indigenous and 1494 non–Indigenous females, respectively [3]. The prevalence of HPV 16/18 in this group was 12.2% among Indigenous and 12.8% among non–Indigenous females, respectively.

### HPV and Oropharyngeal Squamous Cell Carcinoma

Oropharyngeal cancers include cancer of the middle part of the throat: the tonsils, soft palate, and the base of the tongue [4]. HPV-associated oropharyngeal squamous cell carcinoma (OPSCC) has one of the most rapidly increasing incidences in high-income countries [5], with survival rates of HPV-attributable OPSCC being much higher than non–HPV-attributable OPSCC [6]. The increased incidence, which is particularly notable among males and younger cohorts, may be attributable to increased carriage of high-risk HPV types (especially HPV 16 or 18) via increased oral exposure to infected anogenital sites with changing sexual behaviors, as opposed to the more traditional risk factors such as smoking and alcohol [7,8]. Although estimates vary by setting, the proportion of OPSCC attributable to HPV (mainly HPV 16) has been cited in high-income countries as between 65% and 83% [9-11]. OPSCC has now surpassed cervical cancer as the most common HPV-driven cancer [12,13].

### Burden of OPSCC in Australia

In 2008, age-standardized rates of lip, oral cavity, and oropharyngeal cancer in Australia were 11.0 per 100,000 for males and 4.1 per 100,000 for females [14]. Overall rates of overall lip, oral cavity, and oropharyngeal cancer declined between 1982 and 2008, but when considered in isolation, OPSCC rates increased during this time (1.2% per annum for males and 0.8% per annum for females), possibly due to an increased incidence of oral HPV infection [14]. Hocking and colleagues [15] similarly found a significant increase in OPSCC in Australia between 1982 and 2005, with an annual percentage increase of 1.4% for males and 1.0% for females. Hong and colleagues [16] reported that the proportion of OPSCCs that were positive for HPV DNA and p16 increased in males from 19% in 1987-1990 to 47% in 2001-2005 and 60% in 2005-2006. In 2 Brisbane-based studies, the HPV prevalence in patients with OPSCC diagnosed from 2004-10 was 49% and increased to 72% in patients diagnosed from 2013-15 [17,18].

There is little documented evidence of the incidence of OPSCC among Australian Indigenous persons. Indigenous persons living in Queensland were more likely than the total Queensland population to be diagnosed with head and neck cancers between 1997 and 2012, specifically in the base of the tongue/tonsil/oropharynx; the standardized incidence ratio was 2.16 (n=81) [18]. The incidence of oral cavity and oropharyngeal cancer among Aboriginal South Australians between 1977 and 2001 was 29.7 annual cases per 100,000 compared with 8.5 per 100,000 in the non-Aboriginal population [19]. It was the fourth most common cancer type in Aboriginal women, after lung cancer, gynecology-related cancer, and breast cancer. Between 1991 and 2005 in the Northern Territory, oral cavity and oropharyngeal cancer incidence for Indigenous Australians was 2.5 times that of the general Australian population, with the mortality rate being 4.8 times higher in Indigenous men and 4.0 times higher in Indigenous women [20]. In 2015, the rate ratio of disability-adjusted life years due to oral cavity and oropharyngeal cancer among Indigenous Australians was 3.8 times that reported for the total Australian population [21].

### Early Detection of OPSCC

HPV 16-E6 antibodies are detectable in peripheral blood before diagnosis in the majority of HPV 16–driven OPSCCs. Using data from 743 incident OPSCC cases and 5814 controls from 9 population groups across Australia, North America, and Europe, Kreimer and colleagues [13] reported that HPV 16-E6 seropositivity increased with lead time: 0%, 13.5%, 23.7%, and 38.9% with lead times of >30 years (n=24 cases), 20-30 years (n=148), 10-20 years (n=228), and <10 years (n=301), respectively (*P*<.001). Of the 47 HPV 16-E6 seropositive cases with serially collected samples, 17 cases seroconverted during follow-up, ranging from 6 to 28 years before diagnosis. For the remaining 30 cases, robust seropositivity was observed up to 25 years before diagnosis. The authors concluded that the immune response to HPV 16–driven tumorigenesis is detectable several decades before OPSCC diagnosis.

### Accuracy of Saliva Samples for the Detection of Early-Stage OPSCC

Saliva covers the oropharyngeal mucosa and can be collected noninvasively, making it an ideal bodily fluid for early biomarker detection of HPV-induced OPSCC. Recent studies have examined HPV mitochondrial RNA (mRNA) in oral (oropharyngeal swab or saliva) samples from patients with head and/or neck squamous cell carcinoma. The high rates for HPV E6 oncoproteins and E6/E7 mRNA suggest that most patients were experiencing transcriptionally active HPV-related OPSCC [22,23]. The clinically validated Hologic Aptima assay gives a result for the presence of HPV E6/E7 mRNA for 14 HPV types (16, 18, 31, 33, 35, 39, 45, 51, 52, 56, 58, 59, 66, and 68). There is also an option to examine whether the specific HPV type is HPV 16 or HPV 18/45. As with the findings from Kreimer and colleagues [13], the persistence of HPV 16 is a precursor for OPSCC.

### The Indigenous Australian HPV Cohort Study

The first phase of this prospective longitudinal cohort study was developed in partnership with Aboriginal communities in South Australia and funded by the National Health and Medical Research Council in 2016. The study is governed by an Aboriginal Reference Group, with data collected by trained Aboriginal research officers [24]. To be eligible at baseline, participants needed to identify as being Aboriginal and/or Torres Strait Islander, aged 18+ years, and residing in South Australia. Participants were recruited from February 2018 to January 2019 across 8 South Australia sites, primarily through Aboriginal Community Controlled Health Organisations (ACCHOs) whose chief executive officers are key stakeholders. The 1011 participants recruited at baseline represented 5% (1011/20,220) of Aboriginal South Australian adults eligible during the recruitment period, 8.2% (633/7720) of those eligible in nonmetropolitan locations, and 3% (376/12,533) of those in metropolitan locations. Participants were followed-up at 12 months (March 2019 to March 2020), with data from 749 (74.1%) out of 1011 participants obtained (suspended early due to COVID-19 restrictions). Follow-up at 24 months ceased in December 2021, with data from 815 participants obtained. Across baseline and 12- and 24-month follow-ups, saliva samples to test for oral HPV infection were collected in commercially available kits, and the DNA was tested for HPV detection.

### Study Aims

In continued partnership with Aboriginal South Australian stakeholders and study participants, the aims of the second phase of the Indigenous Australian HPV cohort study are as follows:

Aim 1: To extend follow-up to a minimum of 7 years post recruitment to describe the prevalence, incidence, clearance, and persistence of oral HPV infection, including high-risk types, across 48-, 60-, and 72-month follow-ups and to correlate with lifestyle and sociodemographic characteristics

Aim 2: To conduct a thorough clinical examination of the head and neck, oral cavity, and oropharynx and to collect anthropometrics and saliva samples for OPSCC testing

## Methods

### Study Design

**Figure 1:**
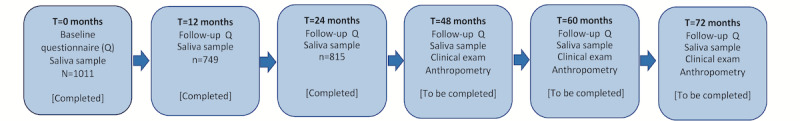
Timing of data and sample collection. T: time point.

We will continue to implement a longitudinal design for the next study phase. A study schema is included in Figure 1.

### Inclusion Criteria and Retention

All participants initially recruited will be eligible for the 48-, 60-, and 72-month follow-ups. Retention strategies will involve those successfully used thus far: (1) continuing to employ Aboriginal staff who have built up personal relationships with participants and who are committed to following up participants despite challenges in doing so; (2) ensuring participants are contacted regularly to ensure the accuracy of contact details; (3) ascertaining contact details of 3 new key personnel who may know the whereabouts of participants should the study team be unable to contact them; (4) sending birthday and Christmas cards to participants; and (5) facilitating one-on-one relationships between study staff and participants, with study staff ideally seeing each of their participants for each research phase. We perceive no difficulties in continuing with the high retention of participants given our past success.

### Ethical Approval

Ethics approval has been received by the Aboriginal Health Council of South Australia’s Human Research Ethics Committee (04-22-1001) and the University of Adelaide’s Human Research Ethics Committee. Prior to being recruited, all participants will be required to sign an informed consent form, which includes consent for the authors to publish the findings in the peer-reviewed scientific literature.

### Data Collection

After completing informed consent with the assistance of the Aboriginal research staff, participants will be again asked to provide saliva samples using a commercially available kit from which microbial DNA and mRNA for genotyping will be extracted. This will involve the participant (1) not eating or drinking for 30 minutes prior to collection; (2) removing the funnel lid on the container; (3) spitting until 2 mL of saliva reaches the fill line on the container (this will take 2 minutes); (4) closing the lid and unscrewing the lid from the funnel; and (5) placing the small cap on the tube and shaking the tube for 5 seconds. The saliva samples will be sent to Victorian Cytology Services Ltd for analysis.

A thorough clinical examination of the external head and neck, intraoral cavity, and oropharynx will be undertaken by trained and calibrated dental professionals. Soft and hard tissue characteristics, caries experience, periodontal disease, and gingivitis will be assessed through standardized oral epidemiological examinations used in national oral health surveys. Weight will be measured in duplicate to the nearest 0.1 kg using Seca model 803 scales and averaged. Height will be measured in duplicate to the nearest 1 mm using a Seca model 213 portable stadiometer, using a standard anthropometric procedure. Any pathology detected via the clinical examination or saliva results will be immediately fed back to participants, with referrals to specialist or local primary care services promptly arranged.

Data collection will be at participants’ homes, ACCHOs, or wherever participants feel the most comfortable.

### Data Analysis

In brief, the analysis plan for each aim is as follows:

#### Aim 1: Oral HPV Infection Prevalence, Incidence, Clearance, and Persistence at 48, 60, and 72 Months

Saliva samples will be stabilized at the point of collection using PreservCyt media. Samples will be run on the real-time polymerase chain reaction–based Seegene HPV 28 assay using the Starlet extraction platform and the BioRad CFX96 amplification/detection instrument. The Seegene HPV 28 assay is the benchmark for HPV surveillance [25] due to its high level of automation, which reduces variation in the sensitivity/specificity between specimens processed at different time points. The Seegene HPV 28 assay gives individual HPV type outputs from 28 HPV types (19 high-risk HPV types: 16, 18, 26, 31, 33, 35, 39, 45, 51, 52, 53, 56, 58, 59, 66, 68, 69, 73, and 82; and 9 low-risk HPV types: 6, 11, 40, 42, 43, 44, 54, 61, and 70), including the 14 HPV types defined by the International Agency for Research on Cancer (IARC) as oncogenic, the additional 2 nononcogenic types covered by HPV vaccination (HPV 6 and HPV 11), and β-globin (for DNA integrity check). HPV 13 and HPV 32 are not included in the Seegene HPV assay but the DNA extraction from this assay is able to be run on a separate polymerase chain reaction based on the protocol by Bolis and colleagues [26]. The prevalence, incidence, clearance, and persistence of oral HPV types across all time points from recruitment at an individual level and collectively will be calculated using simple descriptive analysis.

#### Aim 2: Oral Clinical Examinations/Saliva Samples for OPSCC Testing

The prevalence, extent, and severity of dental diseases, including early clinical signs of OPSCC, will be calculated using standard World Health Organization (WHO) oral health benchmarks [27]. The recently completed 2017-18 National Survey of Adult Oral Health data set [28] will enable comparison with both general and Indigenous population-level estimates. General analysis will comprise chi-square and Student *t* test (2-tailed) within the study sample and nonoverlapping 95% CIs when comparing population estimates. Antibodies against HPV 16-E6 will be measured using saliva samples (Aptima HPV assay) [22]. Assay results will be interpreted based on the signal-to-cutoff ratio, with values ≥ 0.5 considered positive.

## Results

Of the 1011 participants recruited, 910 (90%) provided β-globin positive saliva samples (β-globin is a DNA integrity check). Of these 910, 35.1% (n=319) were positive for any HPV infection [29]. This was 15 times the prevalence reported in a study of young non–Indigenouss Australians [30] and 5 times the prevalence reported in a systematic review involving the United States, Brazil, Mexico, and Finland [31]. Antonsson and colleagues [32] described a longitudinal study (0, 6, 12, and 24 months) of 704 people from Brisbane (18-70 years old). They reported an oral HPV infection prevalence of 10.7% (high-risk HPV prevalence of 6.4%) at baseline in 636 people who were β-globin positive. In our study, the most prevalent HPV types at baseline were those associated with Heck disease (multifocal epithelial hyperplasia; 207/910, 23% of the HPV types found)—a relatively benign and rare condition caused by oral HPV 13 or 32 infection that is more prevalent among Indigenous populations [33-35]. Clinically, Heck disease presents as soft, distinct, mostly multiple, and smooth papules or nodules having the same color as the surrounding mucosal epithelium (a “cobblestone” appearance), predominantly affecting the lower and upper labial mucosa and tongue. The condition usually regresses naturally but can be treated by surgical excision or cryotherapy when lesions are aesthetically unpleasing [36]. The next most prevalent HPV types were those associated with OPSCC (HPV 16 or 18; 30/910, 3.3% of the types found) [29,32].

In our Indigenous Australian HPV Cohort study, of the 749 participants retained at 12-month follow-up, 645 (86.1%) provided β-globin positive saliva samples. Of these, 43% (277/645) were positive for any HPV infection. The most prevalent HPV types at 12-months were again those associated with Heck disease (213/645, 33% of the HPV types found), followed by HPV types associated with OPSCC (HPV 16 or 18; 16/645, 2.5%). A total of 588 participants had samples at baseline and 12-month follow-up that were β-globin positive. The prevalence of any oral HPV infection increased from 34% (200/588) to 44% (259/588). This increase was largely due to increases in HPV types 13 or 32 (Heck disease): from 20% (118/588) at baseline to 34% (200/588) at 12 months. The prevalence of HPV 16 or 18 decreased from 3.9% (23/588) at baseline to 2.7% (16/588) at 12 months. The prevalence of high-risk HPV types according to the IARC definition [37] decreased from 8.5% (50/588) at baseline to 7.1% (42/588) at 12 months [38]. A total of 473 participants had samples at baseline and 12- and 24-month follow-up that were β-globin positive. The prevalence of any oral HPV infection was 24.7% (117/473), the prevalence of HPV type 13 or 32 was 15.6% (74/473), and the prevalence of HPV type 16 or 18 was 1.7% (8/473).

Participant follow-up for this study will commence in January 2023. We anticipate the first results to be submitted for publication 1 year following initial recruitment.

## Discussion

### Expected Findings

Our findings have potential to change the way in which OPSCC among Australian Indigenous adults is managed, with desired impacts including cost-savings on expensive cancer treatments; improved nutritional, social, and emotional outcomes; and improved quality of life for both Indigenous adults and the Indigenous community more broadly.

### Approaches to Aboriginal Community Engagement

The strong Aboriginal partnership, engagement, and buy-in of the first study phase were reported against the Consolidated Criteria for Strengthening Reporting of Health Research involving Indigenous Peoples (CONSIDER) statement [39,40]. The success of our community engagement processes was summarized as (1) engaging with ACCHOs as equal partners very early in the research process; (2) having an Aboriginal Reference Group; (3) having ACCHOs actively promote the study; (4) having a flexible agenda that is responsive to broader environment demands; and (5) including Aboriginal capacity building. Specific lessons learned include the need for active and wide Aboriginal community consultation that is initiated early in the research process, strong and sustained Aboriginal capacity building, and an active Aboriginal Reference Group—all of which we will continue to incorporate in the proposed second phase. There are additional benefits in having Aboriginal staff as representatives in each of the field sites. These representatives have been able to build their reputation in a new area of Aboriginal health, with oral HPV infection not being noticeably recognized previously. Australian Indigenous communities now have a contact, with study continuation facilitating improved HPV knowledge for both the ACCHO workforce and broader community. The next phase of this study will continue to implement and strengthen these community engagement strategies.

### Why Is Continuation of This Cohort Important?

By December 2021, study participants had been followed for a median of 3.5 (range 3.0-3.9) years from baseline, allowing the analysis of short-term outcomes including oral HPV infection prevalence, quality of life, health state valuations of OPSCC, and incidence/clearance in the 12-24 months after baseline. There is not, however, sufficient follow-up to assess whether oral HPV infections, especially persistent high-risk HPV infections, lead to early-stage OPSCC and what the attributable risk from HPV will be. Continuing the largest Indigenous HPV cohort in the world to track oral HPV infection and to monitor the early stages of OPSCC is essential to yield critical information to include in the management armamentarium of health and well-being recommendations for Australia’s First Nations. It is especially relevant in light of the imminent rollout of self-sampling HPV testing initiatives in Australia.

### Strengths and Limitations

There are 2 main strengths of the Indigenous Australian HPV Cohort study. The first of these has been the engagement of South Australia’s Aboriginal communities. This has contributed to the excellent recruitment and follow-up rate (approximately 749/1011, 74% to 815/1011, 80.6%), which needs to be taken into context. For example, this study has been undertaken over vast distances (travelling 700 km west, 400 km east, and 800 km north of the city of Adelaide), involving highly disadvantaged participants who have, in the past, not always enjoyed positive research interactions. The fact that over 1000 participants were recruited in less than 12 months demonstrates the widespread community support. The second strength is the multidisciplinary skill set of the team that is Aboriginal led. The main limitation is the lack of clinical examinations, anthropometrics, and samples to detect HPV 16-E6 antibodies that would yield important biomarker estimates, with funding not provided for this in the first 3 waves of the study.

In conclusion, by continuing the follow-up of the Indigenous Australian HPV Cohort, we will generate novel information regarding oral HPV exposure and OPSCC, including a range of cost-effectiveness modelling to ascertain cost-savings of extending HPV vaccination initiatives beyond what is currently available. This information has potential to vastly increase the relatively unknown causes of such dramatic HPV-related OPSCC incidence rates. Over the past 7 years, we have established excellent community buy-in with Aboriginal groups across South Australia. Everything is in place to continue the follow-up of this essential cohort, with findings likely to be game-changing in terms of influencing Indigenous HPV vaccination policy, oral HPV infections, OPSCC, health policy, and clinical guidelines throughout Australia and, indeed, the world.

## Appendix

Multimedia Appendix 1 Peer-review reports from the National Health and Medical Research Council - 2021 Clinical Trials and Cohort Studies - Australian Government (Canberra, Australia).

## Abbreviations

ACCHO: Aboriginal Community Controlled Health Organisation
CONSIDER: Consolidated Criteria for Strengthening Reporting of Health Research involving Indigenous Peoples
HPV: human papillomavirus
IARC: International Agency for Research on Cancer
mRNA: mitochondrial RNA
OPSCC: oropharyngeal squamous cell carcinoma
WHO: World Health Organization

## Data Availability

The data sets generated and/or analyzed during the current study are not publicly available due to privacy issues of the participants. Data are available from the corresponding author on reasonable request.
