# Supplementary material for: The Indigenous Australian Human Papillomavirus (HPV) Cohort Study 2, Continuation for 5 to 10 Years: Protocol for a Longitudinal Study
Source: JMIR Res Protoc. 2023 May 17;12:e44593. doi: 10.2196/44593 (PMC10233440; doi:10.2196/44593)
Supplement: Multimedia Appendix 1 [file resprot_v12i1e44593_app1.pdf]

Australian Government
National Health and Medical Research Council

NHMRC

Application Assessment Summary
2021 Clinical Trials and Cohort Studies

Application ID: 2014696
Chief Investigator A: Ms Joanne Hedges
Administering Institution: The University of Adelaide

Table 1: **Summary of Individual Scores for your Application**

The average score (1-7) provided by peer reviewers who assessed your application against each of the three assessment criteria and the final weighted average score are provided below.

Assessment Criteria (Average)			
	1	Research Quality (40%)	5.154
	2	Significance (40%)	5.769
	3	Team Quality and Capability (20%)	6.000
		**Overall Score**	**5.569**
		**Category**	**6**

The **funding cut-off** for Clinical Trials and Cohort Studies Grant applications was 5.320, within Category 5 (not including applications funded through structural priority funding).

Note: *For applications deemed Not for Further Consideration, the scores provided in the table above are based on the initial assessments undertaken by the spokesperson assigned to the application. For applications that progressed through to full review at the Grant Review Panel (GRP) meeting, the scores provided above are the final GRP scores.*

Table 2: Summary of Scores for 2021 Clinical Trials and Cohort Studies Grant Applications

Category and average scores for each of the three assessment criteria for applications that proceeded to GRP are provided below. The overall funded rate for Clinical Trials and Cohort Studies Grant applications in 2021 was 11.3%.

Category	No. of applications	S	RQ	TQ&C
7	1	6.909	6.818	6.909
6	17	5.808	5.494	6.048
5	71	5.098	4.594	5.664
4	13	4.305	3.904	5.048

Note:

There were no applications assessed during GRPs that fell within categories 1 2 or 3.

The table above shows the number of applications that were assessed at the GRP meetings for each category.

This table excludes applications that did not request NHMRC funding, and also excludes applications that were deemed ‘Not For Further Consideration’.

Table 3: **Peer Reviewer Comments**

Comments from peer reviewers are provided verbatim, in accordance with the intent of NHMRC's independent expert peer review processes. Comments reflect the assessment of individual peer reviewers and are provided to support applicants' understanding of how their application was scored. NHMRC will respond to complaints about the peer review process but not about the merits of peer reviewer comments. Applicants are encouraged to speak to their Institutional RAO if they are concerned about peer review outcomes.

For more information on the steps NHMRC has taken to support quality peer review, visit: https://www.nhmrc.gov.au/funding/peer-review.

Peer Reviewer Comments:
**Assessor Role:** Spokesperson 1 (Lead)<br>**Question:** Significance Justification<br><br>The proposal is well-placed to address the objective to characterize burden of HPV. Some concern re estimation of burden of HPV-related OPSCC given only 2.5% prevalence of high-risk HPV types in currently established cohort. Presents a great opportunity to build on established cohort and further develop international collaboration. The outcomes should lead to considerable health gains for Aboriginal and Torres Strait Islander people, including beyond the life of the project.
**Assessor Role:** Spokesperson 2<br>**Question:** Significance Justification<br><br>The proposed study is led by an Indigenous researcher along with 2/3rds of team members representing Indigenous communities. Further, the study's Indigenous Reference Group, comprising 9 respected Indigenous adults with diverse backgrounds, has been involved in the design, governance, and general oversight of all phases of the study. The study design and proposed methodology present a unique opportunity to strongly address the higher rates of OPSCC among Indigenous relative to non-Indigenous Australians. This is the largest Indigenous HPV cohort globally, and the follow-up of this cohort at this stage is critical to track oral HPV infection, monitor early stages of OPSCC, and estimate the cost-effectiveness of extensive HPV vaccination coverage in culturally safe ways to improve the health and wellbeing recommendations for Australia's First Peoples.
**Assessor Role:** Spokesperson 3<br>**Question:** Significance Justification<br><br>The outcomes of the study will have a significant health benefit for Aboriginal and Torres Strait Islander peoples, the study will lead to effective health gains for Aboriginal and Torres Strait Islander peoples, beyond the life of the project.
**Assessor Role:** Spokesperson 1 (Lead)<br>**Question:** Research Quality Justification<br><br>The overall research methodology is generally very sound. Only major concern is power to determine burden of HPV-related OPSCC given low prevalence of HPV 16 & 18 types (2.5%) in initial phase. Loss to follow-up of around 30% in years 1 and 2, is reasonable given the setting of the study, but could increase over subsequent 3 years. Strategies to optimize follow-up would need to be closely monitored to ensure that overall follow-up does not fall below 500 over the 5-6 years of the cohort study. Outstanding level of community engagement, which provides enhanced feasibility. In fact, the level of community engagement is a model for Australian Indigenous health research.
**Assessor Role:** Spokesperson 2<br>**Question:** Research Quality Justification<br><br>Outstanding levels of community engagement with demonstrated feasibility.

Assessor Role: Spokesperson 3
Question: Research Quality Justification

The proposal has a research plan that has very good levels of community engagement, ensuring that the proposal is likely to be feasible; and clearly demonstrates how the research and potential outcomes are a priority for the community.

Assessor Role: Spokesperson 1 (Lead)
Question: Team Quality and Capability Justification

The proposal team is an outstanding groups of CIs, with considerable experience and expertise and level of community understanding and engagement. Although CIA does not have a strong academic track record they are clearly well-placed to lead the project, particularly to ensure high-level community engagement. CIB has a particularly strong track record, and presents as an ideal ongoing mentor for CIA. Inclusion of five Aboriginal Medical Service CEOs, although not having strong academic track records, will ensure close community engagement and provides appropriate balance within the CI group. Overall, the CI team includes well-credentialed academics and Aboriginal CEOs, with 2/3 CIs Aboriginal. The proposal will definitely build outstanding capability among Aboriginal and Torres Strait Islander peoples.

Assessor Role: Spokesperson 2
Question: Team Quality and Capability Justification

The team has an excellent track record in working with communities and building capability among Aboriginal and Torres Strait Islander peoples. Further, they have the expertise and experience in cohort study methodology to successfully conduct the proposed study.

Assessor Role: Spokesperson 3
Question: Team Quality and Capability Justification

The team has an excellent track record in working with communities and building capability among Aboriginal and Torres Strait Islander people; it will build excellent capability among Aboriginal and Torres Strait Islander people.

Assessor Role: Spokesperson 1 - Comment Only
Question: Meeting Comments

This is a very strong application with major strengths in relation to addressing an important public health issue, outstanding community engagement and participation , and excellent potential for capacity development. The international collaborative component is a further major strength. The major limitation is around the potential loss to retention of participants, particularly over a six year follow-up period (further 3-4 years in Cohort 2), and the power calculation which was based on overall prevalence of HPV rather than prevalence of high-risk subtypes or incident OPSCC events. A relatively small number of OPSCC events is expected (around 20), based on 600-700 participants followed for 6 years.
